# Cerebral response to emotional working memory based on vocal cues: an fNIRS study

**DOI:** 10.3389/fnhum.2023.1160392

**Published:** 2023-12-29

**Authors:** Saori Ohshima, Michihiko Koeda, Wakana Kawai, Hikaru Saito, Kiyomitsu Niioka, Koki Okuno, Sho Naganawa, Tomoko Hama, Yasushi Kyutoku, Ippeita Dan

**Affiliations:** ^1^Applied Cognitive Neuroscience Laboratory, Faculty of Science and Engineering, Chuo University, Bunkyo, Japan; ^2^Department of Neuropsychiatry, Graduate School of Medicine, Nippon Medical School, Bunkyo, Japan; ^3^Department of Mental Health, Nippon Medical School Tama Nagayama Hospital, Tama, Japan; ^4^Department of Medical Technology, Ehime Prefectural University of Health Sciences, Iyo-gun, Japan; ^5^Department of Clinical Laboratory Medicine, Faculty of Health Science Technology, Bunkyo Gakuin University, Tokyo, Japan

**Keywords:** emotional judgment, voice, n-back, working memory, functional near-infrared spectroscopy, VLPFC, dorsal attention network

## Abstract

**Introduction:**

Humans mainly utilize visual and auditory information as a cue to infer others’ emotions. Previous neuroimaging studies have shown the neural basis of memory processing based on facial expression, but few studies have examined it based on vocal cues. Thus, we aimed to investigate brain regions associated with emotional judgment based on vocal cues using an N-back task paradigm.

**Methods:**

Thirty participants performed N-back tasks requiring them to judge emotion or gender from voices that contained both emotion and gender information. During these tasks, cerebral hemodynamic response was measured using functional near-infrared spectroscopy (fNIRS).

**Results:**

The results revealed that during the Emotion 2-back task there was significant activation in the frontal area, including the right precentral and inferior frontal gyri, possibly reflecting the function of an attentional network with auditory top-down processing. In addition, there was significant activation in the ventrolateral prefrontal cortex, which is known to be a major part of the working memory center.

**Discussion:**

These results suggest that, compared to judging the gender of voice stimuli, when judging emotional information, attention is directed more deeply and demands for higher-order cognition, including working memory, are greater. We have revealed for the first time the specific neural basis for emotional judgments based on vocal cues compared to that for gender judgments based on vocal cues.

## 1 Introduction

When we communicate with others, we mainly use visual and auditory information as a cue to infer the emotions of others. Visual information includes facial expressions, gestures, eye contact, and distance from others, while auditory information includes the voices of others (Gregersen, 2005; Esposito et al., 2009). In general, humans are more highly evolved to detect and process visual information from faces than are other species. It has been shown that we prefer facial information or face-like patterns to other visual information or nonface-like patterns and look at such patterns for longer periods of time (Morton and Johnson, 1991; Valenza et al., 1996). Thus, facial expressions are considered to be major cues for inferring the emotions of others (Neta et al., 2011; Neta and Whalen, 2011).

On the other hand, it has been pointed out that the effectiveness of voice for inferring emotions cannot be ignored because it accurately reflects people’s intended emotions (Johnstone and Scherer, 2000). For example, we can interpret that the speaker is happy when hearing a bright, high-pitched voice, or that the speaker is afraid when hearing a screeching, high-pitched voice. There are several studies focusing on the influence of voice on the inference of emotions (De Gelder and Vroomen, 2000; Collignon et al., 2008). De Gelder and Vroomen (2000) showed interaction between visual and auditory information while inferring emotions, using voices and pictures expressing happy or sad emotions. They confirmed that inferences of emotions depicted in pictures were more accurate when the emotions in the pictures matched those of the vocal stimuli than when they did not match. Collignon et al. (2008) prepared voice and facial expression stimuli depicting two emotions, fear and disgust. Then, they presented them alone or in combination, and asked participants to infer the emotions depicted. Participants inferred emotions more quickly and more accurately when voice and facial expressions containing congruent emotions were presented than when either one of them was presented alone. Thus, the voice also works as a complementary cue for inferring other people’s emotions, and it is believed that accurate and faster decisions can be made when auditory information is added to visual information.

Recent studies on the physiological mechanisms behind inferring emotions by focusing on vocal stimuli have been conducted using functional magnetic resonance imaging (fMRI). For example, Koeda et al. (2013a) used fMRI to examine the brain regions associated with emotion inference. They compared the brain activation during the inference of emotions in healthy subjects with that of schizophrenic patients. In general, schizophrenic patients have more difficulty inferring emotions than do healthy subjects, which leads to difficulty in communicating (Bucci et al., 2008; Galderisi et al., 2013). The voice stimuli in their study were presented in the form of greetings such as “hello” and “good morning” uttered by males or females with positive, negative, or no emotion. Participants were asked to infer the emotion or gender depicted in each voice stimulus. Greater activation of the left superior temporal gyrus in healthy subjects was observed compared to that of schizophrenic patients. Thus, they concluded that the left superior temporal gyrus is relevant when inferring emotions from voices. Koeda et al. (2013a) study focused mainly on the aspects of semantic processing of emotions. In other words, they focused on the discrimination of specific emotions.

Moreover, other research focusing on the aspect of cognitive mechanisms related to the inference of emotions has also been conducted using facial expressions as visual stimuli (Neta and Whalen, 2011). Models for face perception assume a difference between the neural circuits that support the perception of changeable facial features (e.g., emotional expression, gaze) and of invariant facial features (e.g., facial structure, identity) (Haxby et al., 2000; Calder and Young, 2005). For example, within the core system of the cognitive processing of faces, it has been shown that changeable features elicited greater activation in the superior temporal sulcus, and invariant features elicited greater activation in the lateral spindle gyrus (Haxby et al., 2000). Furthermore, in conjunction with this core system, it has been shown that activation in other regions that extract relevant meanings from faces (e.g., amygdala/insula for emotional information, intraparietal sulcus for spatial attention, and auditory cortex for speech) was also observed (Haxby et al., 2000).

To understand the processing in inference of emotions, one study asked subjects to retain information about faces in working memory (Neta and Whalen, 2011). Working memory is an essential component of many cognitive operations, from complex decision making to selective attention (Baddeley and Hitch, 1974; Baddeley, 1998). Working memory is commonly examined using an N-back task, in which participants are asked to judge whether the current stimulus matches the stimulus presented N-stimuli before. Importantly, the N-back task has been used in some studies on face processing (Hoffman and Haxby, 2000; Braver et al., 2001; Leibenluft et al., 2004; Gobbini and Haxby, 2007; Weiner and Grill-Spector, 2010).

The N-back task has also been used to identify neuroanatomical networks, respectively, related to changeable facial features (e.g., emotional expression) and invariant facial features (e.g., identity) (e.g., Haxby et al., 2000; Calder and Young, 2005). LoPresti et al. (2008) confirmed that the sustained activation of the left orbitofrontal cortex was greater during an emotion N-back task than during an identity N-back task using faces as stimuli. In addition, transient activations of the temporal and occipital cortices, including the right inferior occipital cortex, were greater during the identity N-back task. On the other hand, those of the right superior temporal sulcus and posterior parahippocampal cortex were greater during the emotion N-back task.

Based on these findings, some studies have focused on the neural processing of the inference of emotions from facial expressions, which are changeable features of faces. Neta and Whalen (2011) used fMRI to evaluate brain activation during two types of 2-back tasks, using the standard design. During the tasks, participants were required to judge whether the emotion or gender depicted by the current stimulus was the same as that of the stimulus presented 2 stimuli back. The results showed that the activations in the right posterior superior temporal sulcus and the bilateral inferior frontal gyrus during the Emotion 2-back task were significantly greater than those during the Identity 2-back task. In contrast, the rostral/ventral anterior cingulate cortex, bilateral precuneus, and right temporoparietal junction were significantly more active during the Identity 2-back task than during the Emotion 2-back task. Moreover, participants who exhibited greater activation in the DLPFC during both tasks also exhibited greater activation in the amygdala during the Emotion 2-back task and in the lateral spindle gyrus during the Identity 2-back task than those who had less activation in the DLPFC. Interestingly, the activation levels of the DLPFC and amygdala/spindle gyrus were significantly correlated with behavioral performance factors, such as accuracy (ACC) and reaction time (RT), for each task. Based on these findings, Neta and Whalen (2011) postulated that the DLPFC acts as a part of the core system for working memory tasks in general.

On the other hand, it has been suggested that not only the DLPFC but also the VLPFC may relate to WM (Braver et al., 2001; Kostopoulos and Petrides, 2004). In particular, Braver et al. (2001) revealed that the right VLPFC worked selectively for processing non-verbal items such as unfamiliar faces. It should be noted that the bilateral VLPFC was recruited in the Emotion 2-back task as mentioned above (Neta and Whalen, 2011).

Although the neural bases of the cognitive process related to inference of emotions based on facial expression have become clearer, those for vocal cues have not to date been clarified despite their importance. In fact, often we are forced to infer the emotions of others correctly based only on auditory information, not visual information (e.g., during a telephone call). In the processing of inferred emotions based on vocal cues, the DLPFC and VLPFC should be involved as parts of the core system of working memory, as with visual cues (Braver et al., 2001; Neta and Whalen, 2011). However, the findings the Braver et al. (2001) and Neta and Whalen (2011) studies are not directly generalizable due to differences in the sensory modalities. In other words, the inference of emotions based on vocal cues might involve cognitive components specific to auditory processing.

Therefore, the purpose of our study was to clarify the neural basis of processing related to inferring emotions based on vocal cues using the N-back task paradigm. We named one version of the N-back task, in which participants were asked to judge the emotion depicted by the stimuli, the Emotion task, and, the other version of the task, in which they were asked to judge the gender depicted, the Identity task. In addition, as did Neta and Whalen (2011), we examined whether greater activation in specific brain regions was associated with behavioral performance advantages while inferring emotions based on vocal cues.

Moreover, in order to examine differences in judgment strategies between the Emotion and Identity tasks, we also examined the correlations between the behavioral performance for each. For example, if it were shown that the faster the RT, the higher the percentage of correct answers (i.e., negative correlation), then it would be possible that responding quickly leads to better performance. In such a case, there would be no need for cognitive control to be applied to the automatized process. On the other hand, if it were shown that the slower the RT, the higher the percentage of correct responses (i.e., positive correlation), then careful responses may be associated with better performance. In such a case, the inhibitory function would be required, and the cognitive demand associated with recognition should increase.

We utilized functional near-infrared spectroscopy (fNIRS) to measure brain functions. Although fNIRS cannot measure the deep regions of the brain, it has advantages that fMRI does not. fNIRS can measure cerebral hemodynamic responses caused by brain activation relatively easily by simply attaching a probe to the subject’s head. Notably, fNIRS measurement equipment is less constraining and is relatively quiet. It does not require a special measurement environment. fMRI, however, unavoidably creates a noisy environment, which is not conducive to processing emotion judgments based on vocal cues. Thus, fNIRS was judged to be the best method to achieve the purpose of this study.

## 2 Methods

### 2.1 Participants and ethics

Thirty right-handed, healthy volunteers (13 males and 17 females, mean age 21.83 ± 0.97 years, range 20–24) participated in this study. All participants were native Japanese speakers with no history of neurological, psychiatric, or cardiac disorders. They had normal or corrected-to-normal vision and normal color vision. Handedness was assessed by means of the Edinburgh Inventory (Oldfield, 1971). This study was approved by the institutional ethics committee of Chuo University, and the protocol was in accordance with the Declaration of Helsinki guidelines. Two participants were removed from the sample due to unavailability for complete experimental data. As a result, the final sample contained 28 participants.

### 2.2 Experimental procedure and stimulus

**Figure 1:**
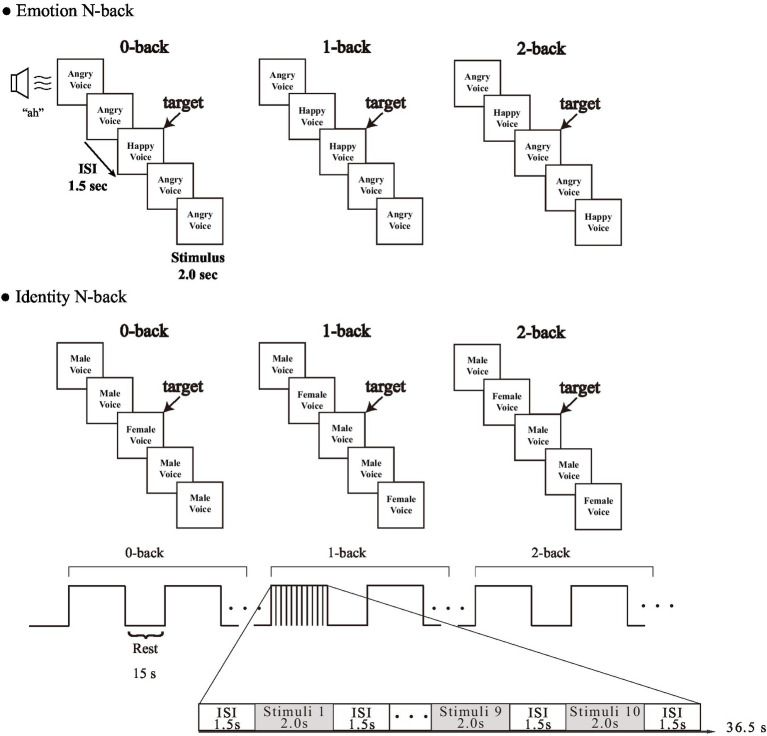
Experimental design of each task.

Participants performed an Emotion task to judge emotional information and an Identity task to determine gender information as in a previous study on facial expression (Neta and Whalen, 2011). We used two audio sets for the emotion condition. The first expressed specific emotional vocalizations by Canadian actors: “Anger,” “Disgust,” “Fear,” “Pain,” “Sadness,” “Surprise,” “Happiness,” and “Pleasure” (Montreal Affective Voices: MAV) (Belin et al., 2008). The second was created using Japanese actors with the same eight emotions expressed. We named it Tokyo Affective Voices (TAV). Simple “ah” sounds were used as a control for the influence of lexical-semantic processing (Figure 1).

According to Koeda et al. (2013b), when Japanese participants listened to the MAVs, they recognized the positive emotion “Happy” and the opposite negative emotion “Angry” with high ACC. Therefore, we used these for the vocal stimuli; we selected two emotions, “Happy” and “Angry,” expressed by both “Male” and “Female” voices from both MAV and TAV, resulting in eight vocal stimuli.

The task load consisted of 0-back, 1-back, and 2-back. In accordance with Nakao et al. (2011) and Li et al. (2014), we performed 0-back, 1-back, and 2-back in sequence, in order to avoid the effect of tension caused by a sudden increase in load, for example jumping from 0-back to 2-back. Each condition was repeated twice for a total of 12 blocks. First, participants performed an N-back task in which they were asked to retain either the emotion (emotional information) or the identity (gender information) in their working memory. Second, in the 0-back condition, participants were asked to respond to the target emotion or gender by pressing a particular key on a keyboard, and a different key for non-targets. Subsequently, the participants were asked to judge whether the current emotion or gender being presented was the same or not the same as the emotion or gender presented immediately before (1-back) or two trials (stimulus presentations) before (2-back) by pressing a key as in the 0-back condition.

We presented each emotion (“Happy” and “Angry”) and each gender (“Male” and “Female”) in each type of voice (MAV and TAV) the same number of times. Each block condition included 10 trials (4 target trials and 6 non-target trials) in random order, each being presented once per block for 2 s, with an inter-stimulus interval of 1.5 s, and a 15-s blank between conditions. Before the start of a new block, the word “0-back,” “1-back,” or “2-back” appeared in the center of the screen to indicate to the participant which task they should prepare to perform. The participants answered by pressing “F” or “J” on a keyboard with their left and right index fingers, respectively, to indicate whether the presented voices were target or non-target trials. We used E-prime 2.0 (Psychology Software Tools) to create these tasks. ACC and RT were obtained as behavioral measures for each trial.

Conventionally, when the distance from the speaker to the participant’s ear is 1 meter, the sound pressure is 65 dB (Shiraishi and Kanda, 2010). As each stimulus had a different sound pressure, we set the highest sound pressure at 65 dB. We used NL-27 (RION Corporation, Kokubunji, Japan) as the sound pressure meter. It was mounted at a height of 138.5 cm above the ground and at a distance of 118 cm from the speaker. Stimuli were presented using a JBL Pebbles speaker (HARMAN International Corporation, Northridge, CA, United States).

### 2.3 fNIRS measurement

We used a multichannel fNIRS system, ETG-4000 (Hitachi Corporation, Tokyo, Japan), which uses two wavelengths of near-infrared light (695 and 830 nm). We analyzed the optical data based on the modified Beer–Lambert Law (Cope et al., 1988) as previously described (Maki et al., 1995). This method enabled us to calculate signals reflecting the oxygenated hemoglobin (oxy-Hb), deoxygenated hemoglobin (deoxy-Hb), and total hemoglobin (total-Hb) signal changes, calculated in units of millimolar × millimeter (mM × mm). The sampling rate was set at 10 Hz. We used the oxy-Hb for analysis because it is the most sensitive indicator of regional cerebral hemodynamic response (Hoshi et al., 2001; Strangman et al., 2002; Hoshi, 2003).

### 2.4 fNIRS probe placement

We used a 3 × 11 multichannel probe holder that consisted of 17 illuminating and 16 detecting probes arranged alternately at an inter-probe distance of 3 cm. The probe was fixed using one 9 × 34 cm rubber shell and bandages mainly over the frontal areas. We defined channel positions in compliance with the international 10–20 system for EEG (Klem et al., 1999; Jurcak et al., 2007). The lowest probes were positioned along the Fpz, T3, and T4 line (horizontal reference curve).

**Figure 2:**
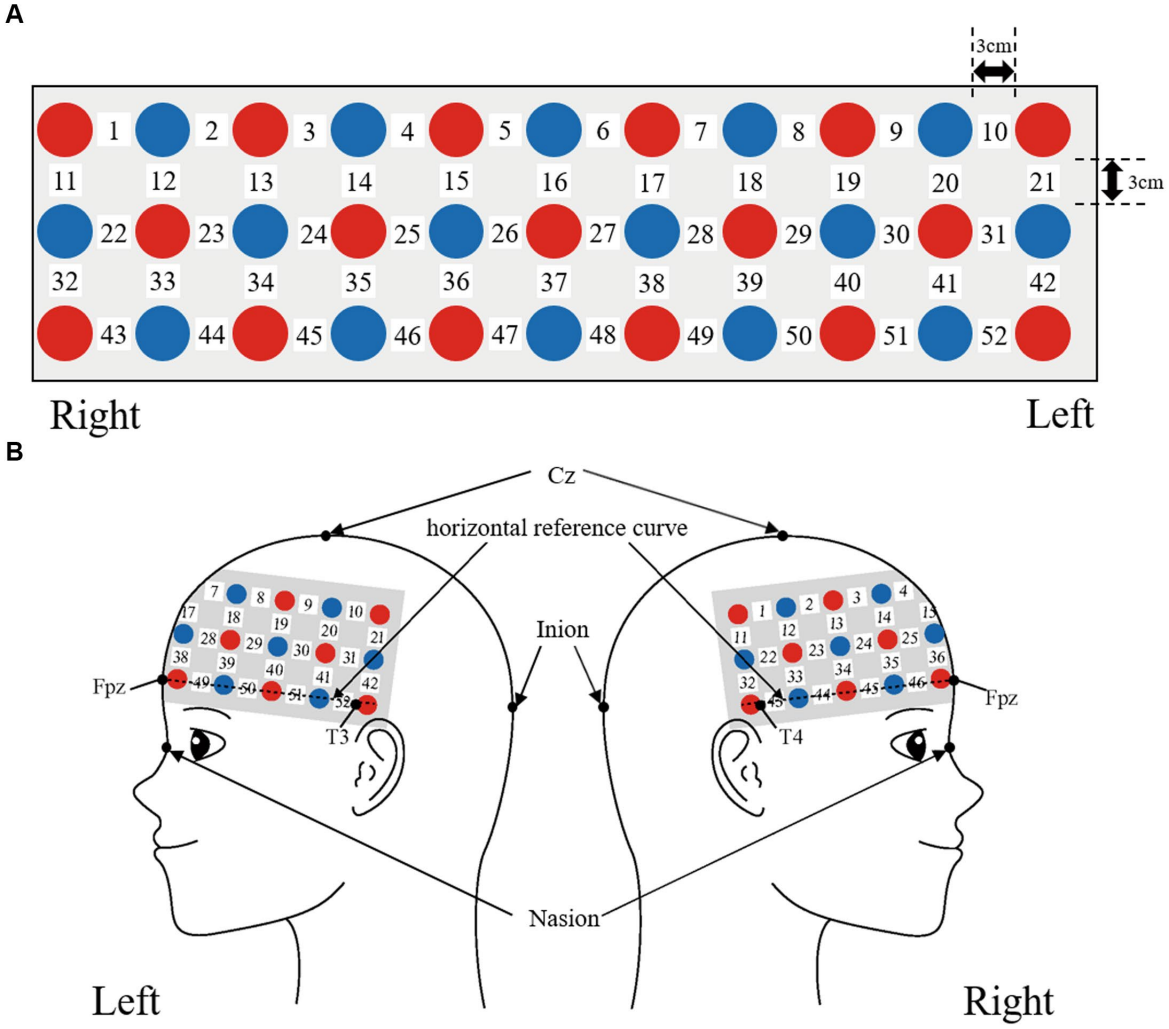
Spatial profiles of fNIRS channels. Detectors are indicated with blue circles, illuminators with red circles, and channels with white squares. **(A)** Position of probes and channels. **(B)** Left and right side views of the probe arrangement are exhibited with fNIRS channel orientation.

**Figure 3:**
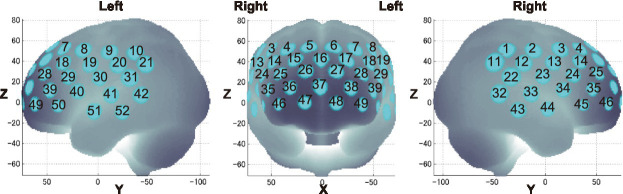
Channel locations on the brain are exhibited in left, frontal, and right side views. Probabilistically estimated fNIRS channel locations (centers of blue circles) and their spatial variability of 8 standard deviation (radii of the blue circles) associated with the estimation are depicted in Montreal Neurological Institute (MNI) space.

Probabilistic spatial registration was employed to register the positions of each channel to Montreal Neurological Institute (MNI) standard brain space (Tsuzuki et al., 2007; Tsuzuki and Dan, 2014). Specifically, the positions for channels and reference points, which included the Nz (nasion), Cz (midline central), and left and right preauricular points, were measured using a three-dimensional digitizer in real-world (RW) space. We affine-transformed each RW reference point to the corresponding MRI-database reference point and then replaced them to MNI space (Okamoto and Dan, 2005; Singh et al., 2005). Adopting the same transformation parameters enabled us to obtain the MNI coordinate values for the position of each channel in order to obtain the most likely estimate of the location of given channels for the group of participants and the spatial variability associated with the estimation. Finally, the estimated locations were anatomically labeled using a MATLAB^®^ function that reads anatomical labeling information coded in a microanatomical brain atlas, LBPA40 (Shattuck et al., 2008) and Brodmann’s atlas (Rorden and Brett, 2000; Figures 2, 3).

### 2.5 Behavioral performance data

To validate the results of brain activation analyses, we measured ACC and RT for each stimulus during the N-back tasks. Then, ACC and RT means were calculated for each participant. The calculated data were subjected to three-way repeated measures ANOVA, 3 (load: 0-back, 1-back, 2-back) × 2 (stimulus: Emotion, Identity) × 2 (trial: target, non-target), using IBM SPSS Statistics 26. For main effects, Bonferroni correction was performed. The significance level was set at 0.05.

### 2.6 Preprocessing of fNIRS data

We used Platform for Optical Topography Analysis Tools (POTATo) (Sutoko et al., 2016) for data preprocessing. Individual participants’ timeline data for the oxy-Hb signal of each channel were preprocessed with a first-degree polynomial fitting and high-pass filter using cut-off frequencies of 0.0107-Hz to remove baseline drift, and a 0.1-Hz low-pass filter to remove heartbeat and pulse. We fit the baseline for task blocks with averages of 10 s before the introduction period.

### 2.7 fNIRS analysis

From the preprocessed time series data, we computed channel-wise and subject-wise contrasts by calculating the inter-trial mean of differences between the oxy-Hb signals for target periods (13–36.5 s after the start of the N-back task) and baseline periods (10 s before the start of the N-back task). We performed a one-sample *t*-test against zero (two tails) on preprocessed data.

A previous study (Zhou et al., 2021) demonstrated that brain activation is greater when the task load is efficient than when it is excessive or insufficient. Therefore, we explored conditions with the most sufficient cognitive loads to detect brain activations related to the N-back task in our study.

Activated channels were estimated from the effect size. We used G*Power (release 3.1.9.7) to consider a reasonable effect size for the 28 participants (Faul et al., 2007). Power analysis was conducted under the conditions of sample size = 28, one-sample *t*-test with *α* = 0.01 and power = 0.80. A reasonable effect size of 0.57 was obtained to maintain the balance between Type I and Type II errors, based on the study by Cohen (1992). Therefore, we defined a reasonable effect size of 0.57 or more as activation of brain function in this analysis.

### 2.8 Between participants correlations

We conducted Spearman’s correlation analyses for behavioral performance and brain activation to explore whether there was a connection between brain activation and behavioral performance. In addition, we conducted Spearman’s correlation analyses between ACC and RT to examine whether judgment strategy differed between judging emotion and gender. We used IBM SPSS Statistics version 26. The significance level was set at 0.05.

## 3 Results

### 3.1 Behavior results

**Figure 4:**
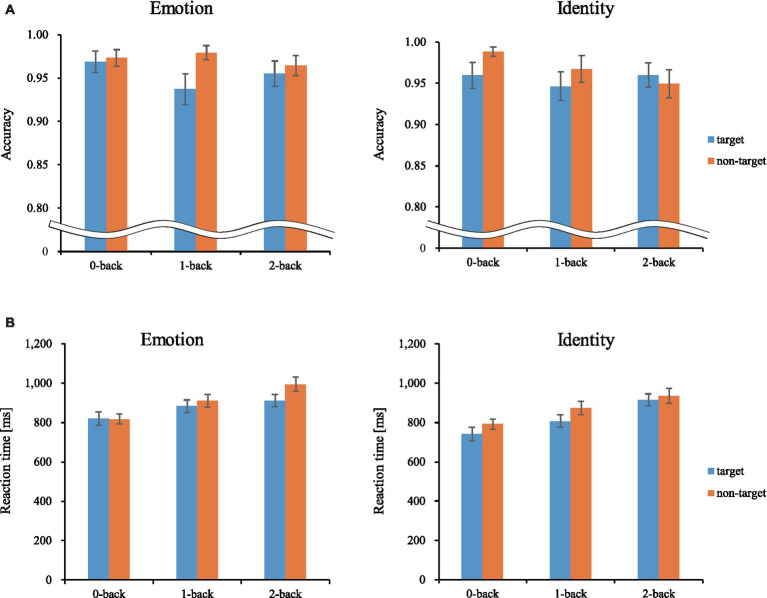
Mean accuracy and reaction time. Error bars represent standard error. ms, millisecond. **(A)** Accuracy. **(B)** Reaction time.

We calculated behavioral performance separately for each load, stimulus, and trial. For both ACC and RT, we conducted a three-way repeated-measures ANOVA, 3 (load: 0-back, 1-back, 2-back) × 2 (stimulus: Emotion, Identity) × 2 (trial: target, non-target). Figure 4 shows the results of the three-way ANOVA.

#### 3.1.1 Accuracy

We revealed a significant main effect of trials [*F*(1, 27) = 10.092, *p* < 0.01, *η_p_*^2^ = 0.272]. Corrected pairwise comparisons (Bonferroni correction) revealed that participants were more accurate for non-target trials than target trials (*p* < 0.01).

#### 3.1.2 Reaction time

We revealed a significant main effect of all factors [loads: *F*(2, 54) = 28.523, *p* < 0.001, *η_p_*^2^ = 0.514; stimuli: *F*(1, 27) = 32.326, *p* < 0.001, *η_p_*^2^ = 0.545; trials: *F*(1, 27) = 18.382, *p* < 0.001, *η_p_*^2^ = 0.405]. There was also a three-way interaction [*F*(2, 54) = 4.364, *p* < 0.05, *η_p_*^2^ = 0.139]. The *post-hoc* test showed that RTs were significantly longer between loads in the following order (longest to shortest): 2-back, 1-back, 0-back. The RTs were also significantly longer for the Emotion task than for the Identity task, and longer for non-target trials than for target trials. These results show that the 2-back task had the lowest ACC and the longest RT of the three loads. Therefore, we used performance data from during the 2-back task, which produced the best possible cognitive loads from among the three conditions, for further analysis (Tables 1, 2).

**Table 1:** Summary of results of activation analysis of Emotion 2-back task.

CH	*M*	*SD*	*t*	Sig	*d*
11	0.037	0.05	4.19	<0.001	0.79
13	0.054	0.09	3.20	0.004	0.60
21	0.035	0.05	3.65	0.001	0.69
24	0.044	0.07	3.26	0.003	0.62
39	0.035	0.05	3.99	<0.001	0.75
42	0.050	0.08	3.33	0.003	0.63
46	0.039	0.06	3.46	0.002	0.65

SD, standard deviation; *t*, *t*-value.

**Table 2:** Summary results of activation analysis for Identity task.

CH	*M*	*SD*	*t*	*p*	*d*
20	0.048	0.080	3.16	0.004	0.60
21	0.035	0.056	3.34	0.002	0.63
30	0.074	0.104	3.77	0.001	0.71
31	0.070	0.110	3.36	0.002	0.63
32	0.054	0.076	3.81	0.001	0.72
42	0.075	0.114	3.48	0.002	0.66
46	0.043	0.059	3.92	0.001	0.74

SD, standard deviation; *t*, t-value.

#### 3.1.3 fNIRS results

**Figure 5:**
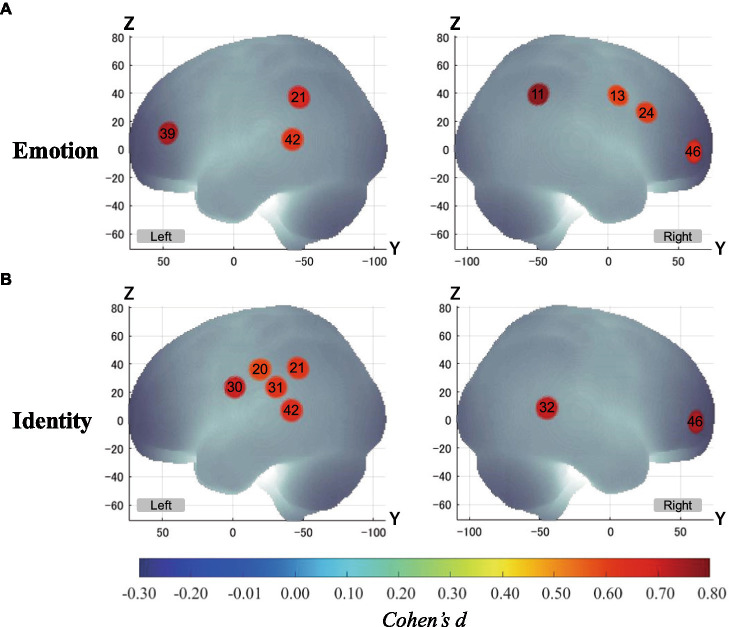
Activation t-maps of oxy-Hb signal increase during 2-back task. Only channels defined as activated (*α* < 0.01 and Cohen’s *d* > 0.57) are exhibited in MNI space. Among them, Ch39 was found activated during the 1-back Emotion task and Chs21 and 32 were activated during the 1-back Identity task. For both 2-back task conditions, all channels indicated above were activated, and no channels were found activated in any of the 0-back tasks. **(A)** Emotion task. **(B)** Identity task.

Figure 5 shows the results of a one-sample *t*-test (vs. 0) on the calculated interval means. Our results showed a significant oxy-Hb signal increase during the Emotion 2-back task in seven channels and during the Identity 2-back task in seven channels. Tables 3, 4 show the locations of channels with significant activation. As task difficulty increased, activated channels increased progressively from the 0-back tasks with no activations, to one channel during Emotion and two channels during Identity 1-back tasks, and, finally, to all the channels indicated in Figure 5 during the respective 2-back tasks.

**Table 3:** Estimated most likely locations of activated channels from spatial registration for Emotion 2-back task.

CH	MNI-coordinates				LPBA40	Prob. (%)	Brodmann area	Prob. (%)
	*x*	*y*	*z*	*SD*				
11	64.7	−48.3	39.7	10.0	R AG	78	SMG part of Wernicke’s area (BA40)	90
13	61.0	7.3	38.3	8.5	R PRG	93	Pre-SMA(BA6)	81
21	−65.0	−46.3	37.3	9.5	L SMG	67	SMG part of Wernicke’s area (BA40)	87
24	57.3	27.7	26.3	7.8	R IFG	87	Pars triangularis Broca’s area (BA45)	85
39	−49.0	46.7	11.7	8.3	L IFG	71	Pars triangularis Broca’s area (BA45)	52
42	−70.0	−41.7	7.3	8.8	L STG	51	STG (BA22)	79
46	42.7	61.3	−1.3	6.8	R IFG	89	FPA (BA10)	74

Prob, probability; SD, standard deviation. SD is expressed in millimeters (mm). AG, angular gyrus; IFG, inferior frontal gyrus; FPA, frontopolar cortex; Pre-SMA, pre-motor and supplementary motor cortex; PRG, precentral gyrus; SMG, supramarginal gyrus; STG, superior temporal gyrus.

**Table 4:** Estimated most likely locations of activated channels from spatial registration for Identity 2-back task.

CH	MNI-coordinates				LPBA40	Prob. (%)	Brodmann area	Prob. (%)
	*x*	*y*	*z*	*SD*				
20	−66.0	−19.3	36.7	8.9	L SMG	72	S1 (BA2)	49
21	−65.0	−46.3	37.3	9.5	L SMG	67	SMG part of Wernicke’s area (BA40)	87
30	−65.7	−1.3	24.3	8.1	L PoCG	57	Subcentral area (BA43)	87
31	−69.0	−30.7	24.3	9.1	L SMG	61	S1 (BA2)	42
32	71.0	−44.3	9.3	9.3	R MTG	76	STG (BA22)	77
42	−70.0	−41.7	7.3	8.8	L STG	51	STG (BA22)	79
46	42.7	61.3	−1.3	6.8	R IFG	89	FPA (BA10)	74

Prob, probability; SD, standard deviation. SD is expressed in millimeters (mm). FPA, frontopolar cortex; IFG, inferior frontal gyrus; MTG, middle temporal gyrus; PoCG, post central gyrus; S1, primary somatosensory cortex; SMG, supramarginal gyrus; STG, superior temporal gyrus.

### 3.2 Between-participant correlations

First, we conducted Spearman’s correlation analysis between ACC and brain activation. There were no significant correlations in the Identity 2-back task (Table 5).

**Table 5:** Correlation between brain activation and ACC during Emotion 2-back task.

CH	MNI-coordinates				LPBA40	Prob. (%)	Brodmann area	Prob. (%)
	*x*	*y*	*z*	*SD*				
4	32.7	33.7	52.0	9.4	R MFG	95	DLPFC (BA9)	66
12	68.0	−20.3	38.7	8.6	R SMG	100	S1 (BA1)	54
13	61.0	7.3	38.3	8.5	R PRG	93	Pre-SMA (BA6)	81
22	71.0	−32.7	26.3	9.5	R SMG	64	S1 (BA2)	31
24	57.3	27.7	26.3	7.8	R IFG	87	Pars triangularis Broca’s area (BA45)	85

Prob, probability; SD, standard deviation. SD is expressed in millimeters (mm). DLPFC, dorsolateral prefrontal cortex; IFG, inferior frontal gyrus; MFG, middle frontal gyrus; Pre-SMA, pre-motor and supplementary motor cortex; PRG, precentral gyrus; S1, primary somatosensory cortex; SMG, supramarginal gyrus.

Second, we conducted Spearman’s correlation analysis between RT and brain activation. There were no significant correlations in the Emotion 2-back task. In the Identity 2-back task, we found significant positive correlations in five channels: channel 20, *ρ*(28) = 0.43, *p* < 0.05; channel 41, *ρ*(28) = 0.47, *p* < 0.05; channel 42, *ρ*(28) = 0.44, *p* < 0.05; channel 51, *ρ*(28) = 0.48, *p* < 0.05; and channel 52, *ρ*(28) = 0.41, *p* < 0.05. There was also a significant negative correlation in one channel: channel 24, *ρ*(28) = −0.43, *p* < 0.05. Table 6 shows the locations of channels with significant correlations.

**Table 6:** Correlation between brain activation signals and RT during Identity 2-back task.

CH	MNI-coordinates				LPBA40	Prob. (%)	Brodmann area	Prob. (%)
	*x*	*y*	*z*	*SD*				
20	−66.0	−19.3	36.7	8.9	L SMG	72	S1 (BA2)	49
24	57.3	27.7	26.3	7.8	R IFG	87	Pars triangularis Broca’s area (BA45)	85
31	−69.0	−30.7	24.3	9.1	L SMG	61	S1 (BA2)	42
41	−67.7	−12.7	7.3	8.7	L STG	66	STG (BA22)	94
42	−70.0	−41.7	7.3	8.8	L STG	51	STG (BA22)	79
51	−62.7	3.7	−8.3	9.0	L STG	84	MTG (BA21)	62
52	−71.0	−23.3	−9.3	7.8	L MTG	100	MTG (BA21)	100

Prob, probability; SD, standard deviation. SD is expressed in millimeters (mm). IFG, inferior frontal gyrus; MTG, middle temporal gyrus; S1, primary somatosensory cortex; SMG, supramarginal gyrus; STG, superior temporal gyrus.

Finally, we conducted Spearman’s correlation analysis between ACC and RT. We found a significant positive correlation for the Emotion 2-back task [*ρ*(28) = 0.375, *p* < 0.05]. There were no significant correlations for the Identity 2-back task.

## 4 Discussion

In our present study, to clarify the cognitive mechanisms for the inference of emotions based on vocal speech, we examined brain activation patterns during Emotion and Identity N-back tasks in combination with fNIRS measurements. Our results reveal different patterns not only in behavioral performance but also in brain activation between the tasks. The non-target trials elicited higher ACC than the target trials in both tasks. The Emotion task required longer RTs than the Identity task. Also, RTs were longer for non-target trials than for target trials, decreasing in order of 2-back, 1-back, and 0-back. Moreover, we observed different cortical activation patterns, possibly reflecting the differences in behavioral variables. During the Emotion task, there was activation in the right inferior frontal gyrus. During the Identity task, we observed activations in the left supramarginal, left postcentral, right middle temporal, right inferior frontal, and left superior temporal gyri.

The behavioral results showed that ACC did not differ across the two tasks or between cognitive loads, whereas the RT increased with increasing cognitive loads. In general, the larger the value of N in the N-back task was, the higher the subjective difficulty and the greater the cognitive load were (Yen et al., 2012). This suggests that the cognitive load of the 2-back task was highest, which led to a greater increase in RT compared to the other N-back tasks. Similar to facial expression experiments by Neta and Whalen (2011) and Xin and Lei (2015), RTs were also significantly longer for the Emotion task than for the Identity task. Therefore, it can be supposed that the inference of emotions generally demands a greater cognitive load than the inference of genders across different modalities.

As for the brain activation results, we focused on the 2-back conditions for both tasks since it required a sufficient cognitive load to effectively elicit brain activation reflecting cognitive processing. The number of activated channels increased progressively from no activations in the 0-back condition to a few in the 1-back condition and to several in the 2-back condition. This pattern serves as a good indication that activations during the emotion task were due not to emotion-related physiological responses but to increased cognitive loads for processing emotion-related information since emotional loads are thought to be similar among corresponding n-back conditions.

Our results revealed distinct cortical regions specifically recruited for either the Emotion or Identity task. The right prefrontal regions in channel 24 exhibited specific focused activation during the Emotion task. The Emotion task should require attention to information about a specific emotion (happy or angry), which requires top-down processing based on voice information such as tone and pitch.

Many theoretical accounts of cognitive control have assumed that there would be a single system that mediates such endogenous attention (Posner and Petersen, 1990; Spence and Driver, 1997; Corbetta et al., 2008). Typically, such a system is referred to as the “dorsal attention network” (DAN) (Corbetta et al., 2008), which is a frontal–parietal network including the superior parietal lobule (SPL), the frontal eye field (FEF) in the superior frontal gyrus, and the middle frontal gyrus (MFG) (Corbetta et al., 2008). It has been shown that a top-down attention process is involved (Kincade et al., 2005; Vossel et al., 2006). DAN has been shown to be elicited not only in attention to visual stimuli (Büttner-Ennever and Horn, 1997; Behrmann et al., 2004; Corbetta et al., 2008), but also to auditory stimuli (Driver and Spence, 1998; Bushara et al., 1999; Linden et al., 1999; Downar et al., 2000; Maeder et al., 2001; Macaluso et al., 2003; Mayer et al., 2006; Shomstein and Yantis, 2006; Sridharan et al., 2007; Wu et al., 2007; Langner et al., 2012).

However, recent studies have confirmed the modality specificity of DAN. For visual attention, it has been speculated that the frontoparietal network, including the SPL, FEF, and MFG, plays an important role (Driver and Spence, 1998; Davis et al., 2000; Macaluso et al., 2003; Langner et al., 2011). However, for auditory attention, a network including the MFG and posterior middle temporal gyrus has been shown to be mainly at work (Braga et al., 2013). The right prefrontal regions activated in the current study’s Emotion task were roughly consistent with the regions shown to make up the attentional network for auditory top-down processing (Braga et al., 2013), although channel 13 was located slightly posterior and channels 24 and 46 slightly inferior, respectively.

The slight differences between our results and those described by Braga et al. (2013) may reflect differences of task demands. Braga et al. (2013) requested participants to detect physical features (i.e., changes of pitch) from stereo, naturalistic background sounds. On the other hand, we asked participants to infer the emotions of others from human voices in the Emotion task. Our task would have involved not only physical feature detection but also other cognitive processes (e.g., considering the mood of others), which would, in turn, involve more complex task demands.

From a different perspective, channels 24 and 39 were located over the ventrolateral prefrontal cortex (VLPFC), which is known to play important roles in working memory (Owen et al., 2005; Barbey et al., 2013). Wagner (1999) indicated that VLPFC activity had a privileged role in domain-specific WM processes of phonological and visuospatial rehearsal. However, Jolles et al. (2010) demonstrated that greater activation in the VLPFC was observed in response to the increasing task demands of a WM task. Indeed, our results suggest that the Emotion task would demand more complex cognitive processes since the RTs for the Emotion task were longer than those for the Identity task. Such differences in task demands may be reflected in the cortical activation in the VLPFC observed only during the Emotion task.

On the other hand, there were several regions specifically activated during the Identity task, in particular the left temporal region, including the left supramarginal gyrus, at channels 20, 30, and 31. The supramarginal gyrus has been shown to be involved in short-term auditory memory (Gaab et al., 2003, 2006; Vines et al., 2006). Moreover, hemispheric differences have been reported: During a short-term auditory memory task, there was greater activation in the left supramarginal gyrus than in the right (Gaab et al., 2006). The results of these previous studies suggest that the left supramarginal gyrus works as a center for auditory short-term memory and also influences the allocation of processing in the auditory domain as part of a top-down system.

The right middle temporal gyrus was also activated only during the Identity task. The middle temporal gyrus is known to play an important role in semantic memory processing (Onitsuka et al., 2004; Visser et al., 2012). Clinically, activation in the right middle temporal gyrus is known to be reduced in schizophrenic patients, and its reduction is associated with auditory verbal hallucinations and difficulty in semantic memory processing (Woodruff et al., 1995; Lennox et al., 1999, 2000). These findings suggest that the right temporal gyrus plays an important role in auditory semantic memory processing.

By interpreting the functions of these brain regions during both tasks, we can begin to integratively explain the physiological mechanisms involved in the inference of emotions based on auditory information. Specifically, when judging emotional information compared to judging the gender of voice stimuli, auditory attention could be directed more deeply. Therefore, the regions responsible for complex cognition, including working memory, would be working together. On the other hand, when judging gender rather than emotional information, the demand of processing physical information from a vocal stimulus would, rather, be greater. It is possible that we are making judgments based on the characteristics of the sound itself, including pitch information. This interpretation is rational because the activation in the supramarginal gyrus was localized to the left hemisphere during the Identity task. Previous studies support the predominance of working memory system requirements for emotional judgments as well as the predominance of speech information processing requirements for gender judgments (e.g., Whitney et al., 2011a,b). The left temporal region at channels 20 and 31 which activated during the Identity task and the left inferior frontal gyrus at channel 39 which activated during the Emotion task have been found to play different roles in semantic cognition. The left temporal region is involved in accessing information in the semantic store (Hodges et al., 1992; Vandenberghe et al., 1996; Hickok and Poeppel, 2004; Indefrey and Levelt, 2004; Rogers et al., 2004; Vigneau et al., 2006; Patterson et al., 2007; Binder et al., 2009; Binney et al., 2010). On the other hand, the left inferior frontal gyrus is involved in executive mechanisms that direct the activation of semantics appropriate to the current task and context (Thompson-Schill et al., 1997; Wagner et al., 2001; Bookheimer, 2002; Badre et al., 2005; Ye and Zhou, 2009). The present Identity 2-back task would have required these cognitive processes.

We also examined the correlation between behavioral performance and brain activation to determine how brain activation associated with voice-based inference of emotions is related to behavioral performance. In the Emotion task, the greater the activation in the right frontal region to the supramarginal gyrus was, the higher the ACC was. This suggests that these regions contribute to correctly inferring emotions based on speech information. Regarding the relationship between RT and ACC, in the Emotion task alone, the slower the RT was, the higher the ACC was. This indicates that there was a trade-off between RT and ACC when inferring emotions. Thus, the Emotion task and Identity task elicited different brain activation patterns under different cognitive strategies derived from different cognitive demands.

For the Emotion task, considering the activation of regions related to the auditory attention network, we submit that the cognitive strategy was not an immediate response to auditory cues, but rather an analytical processing of the features of the auditory information with auditory attention for accurate inferences. On the other hand, for the Identity task, no correlation was found between RT and ACC. This indicates that the ACC was not determined by RT, and accurate inferences could be made without sacrificing speed. We can interpret these results with reference to the findings of a previous study examining facial-expression-based inference of emotions (Neta and Whalen, 2011). That is, changeable features (e.g., emotions) involve more complex judgments requiring relatively more auditory attention, similarly to the visual attention mentioned in Neta and Whalen (2011), than do invariant features (e.g., gender, identity).

**Figure 6:**
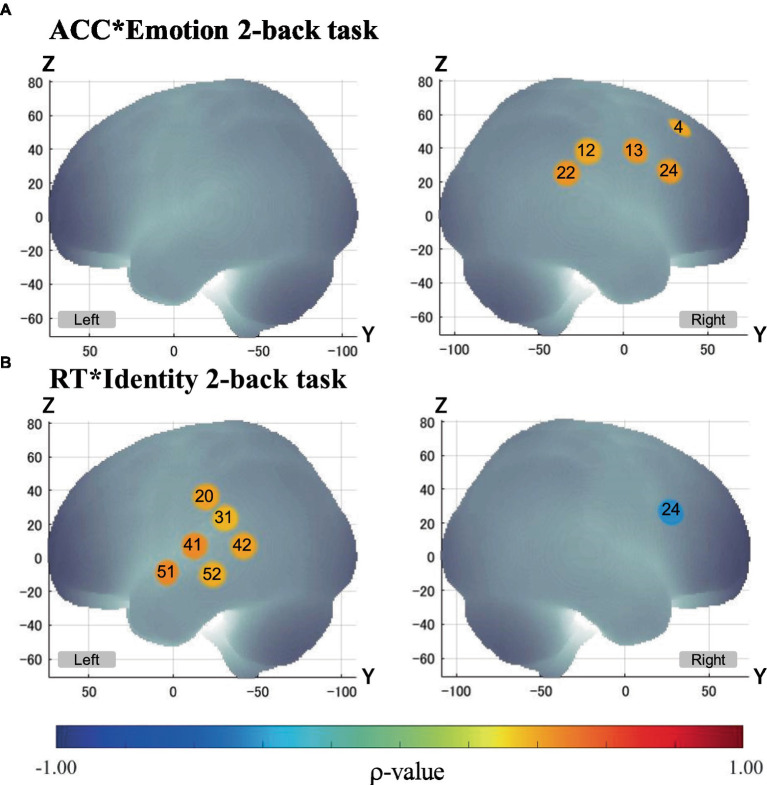
Correlation between brain activation and behavioral performance. Only channels with significant Spearman’s correlation (*α* < 0.05) are exhibited in MNI space. *p* - values are as indicated in the color bar. **(A)** Correlation between ACC and brain activation in the Emotion 2-back task (ACC*Emotion 2-back task). **(B)** Correlation between RT and brain activation in the Identity 2-back task (ACC*Identity 2-back task).

Though N-back tasks are known to require working memory processes (Owen et al., 2005; Kane et al., 2007), the VLPFC in the right inferior frontal regions, which is the center of working memory, elicited activation only during the Emotion task, and not during the Identity task. However, there was a negative correlation between brain activation and RT in the region located on the right VLPFC (channel 24) during the Identity task (see Figure 6). This suggests that the VLPFC served an important function in the Identity task as well as in the Emotion task. Considering the difference in cognitive demands between the Emotion and Identity tasks, it is likely that the demands of speech information processes were more dominant than working memory processes during the Identity task. Thus, no significant activation was observed in the area located on the VLPFC (channel 24) during the Identity task (see Figure 6).

From the results of our study, we can infer that different cognitive demands underlie each task (Emotion and Identity). To determine gender based on voice information, it is sufficient to access the semantic store from the voice features and check whether the feature information is consistent with the knowledge in the semantic store. On the other hand, when judging emotions in speech information, it is necessary not only to check whether the characteristics of the voice are consistent with the emotional information in the semantic store, but also to judge the emotional information in conjunction with the context behind it. In other words, it is necessary to infer the mental state of the speaker by considering not only physically produced qualities such as pitch, but also the context within which the speaker is vocalizing. Such differences in cognitive demand should produce different patterns of brain activation during the Emotion and the Identity N-back tasks. Indeed, during the Emotion task, the reaction time was longer due to the additional processing needed to infer the mental state of the speaker in addition to judging the morphological characteristics of the voice.

In the present study, there are several limitations that should be considered. First, the activation of the amygdala, which is widely known to be related to emotional functioning (e.g., Gallagher and Chiba, 1996; Phelps, 2006; Pessoa, 2010), was not considered; because fNIRS can only measure the activation of the cortical surface, it cannot measure the activation of the amygdala. Since voice-based emotional judgments occur with social interactions, their neural basis is expected to be complex, involving a range of cortical and subcortical areas and connectivity pathways. In the present study, we used speech voices as stimuli, which provide only auditory information. However, as with Neta and Whalen (2011) use of facial expressions as visual information, the amygdala may be involved in emotional judgments regardless of the sensory modality. Though future studies using fMRI may clarify the role of the pyramid in the inference of emotions based on auditory information, there would inevitably be technical problems related to the noisy environment.

Second, the current experimental design did not allow us to discuss the physiological mechanisms that depend on the type of emotion. We aimed to clarify the underlying mechanisms of emotional judgments using two clearly distinguishable emotions (Happy/Angry) measured in the framework of a block design, and we were able to achieve this goal. However, the differences in the relevant domains are unclear due to the differences in the emotional valence. In general, it is known that basic emotions are expressed by two axes, valence and arousal (Yang and Chen, 2012), of which Happy and Angry have equal arousal but significantly different valence (Fehr and Russell, 1984; Yang and Chen, 2012). As a result, Happy is considered a positive emotion and Angry is considered a negative emotion.

In our present study, we used voices with happy and angry emotions as the stimuli; however, there are, of course, other basic emotions, such as sadness, fear, disgust, contempt, and surprise (Ekman and Cordaro, 2011). Different emotions have varied emotional valence and arousal, and such differences could result in different cognitive processing mechanisms. One possible future study could use an event-related design with voices expressing other basic emotions, which would allow us to clarify the mechanisms for each individual basic emotion.

Third, although the current study provides an important step toward the clinical applicability of emotional working memory tasks, we have yet to optimize the analytical methods applied here. Instead of using a regression analysis of the observed time-series data to modeled hemodynamic responses in a time series, a procedure typically referred to as a general linear model (GLM) analysis (Uga et al., 2014), we used a method that averages hemodynamic responses over a fixed time window. This was to ensure robust analyses of observed data with both temporal and spatial heterogeneity. However, to achieve a better understanding of the cognitive processes underlying the emotional and identity n-back tasks, we should optimize either hemodynamic response models, task structures or both.

Fourth, in the current study, we used continuous-wave fNIRS with an equidistant probe setting. However, this entails the possibility of physiological noises such as skin blood flow and systemic signals being included in the observed hemodynamic responses. While such physiological noises tend to be distributed globally, the cortical activation patterns observed in the current study mostly exhibited asymmetric distributions. Thus, it is expected that activations detected in the current study may well represent cognitive components associated with tasks and task contrasts. For further validation, we must extract genuine cognitive components for identity and emotional working memory by reducing the effects of physiological noise to a minimum.

Fifth, in a related vein, data analysis parameters used for the current study were optimized for oxy-Hb data. Had we applied those optimized for deoxy-Hb, it might have led to different results. Although we focused on oxy-Hb results in the current study and reported deoxy-Hb data using the same parameter sets in Supplementary material, future studies might be able to build upon our results and elucidate the potential use of deoxy-Hb signals as they relate to emotion and identity N-back tasks.

Despite the limitations mentioned above, here we have used fNIRS to provide new insights into the neurological mechanisms underlying the inference of emotions based on audio information for the first time. The primary importance of the findings of this study lie in the great potential for clinical applications. Many researchers have shown the association between difficulties inferring emotions and several types of disorders: neurological (Bora et al., 2016a,b), psychiatric (Gray and Tickle-Degnen, 2010; Dalili et al., 2015), and developmental (Bora and Pantelis, 2016). In particular, social cognitive dysfunction, including difficulties inferring emotions based on facial expression cues, has been identified as a potential marker for the developmental disorder autism and the psychopathology schizophrenia (Derntl and Habel, 2011).

Neurodegenerative diseases such as behavioral variant frontopolar dementia (bvFTD) and amyotrophic lateral sclerosis (ALS) have also been found to manifest social cognitive impairment, including difficulties inferring emotions (Bora et al., 2016c; Bora, 2017). Thus, the neurological measurement of such difficulties could serve as a marker for nerve deterioration, disease progression, and even treatment response (Cotter et al., 2016). Cotter et al. (2016) conducted a meta-analysis of related studies and provided a comprehensive review. They concluded that difficulties with the inference of emotions cued by facial expressions could be a core cognitive phenotype of many developmental, neurological, and psychiatric disorders. In addition, though most studies on the relationship between the inference of emotions and disease have used facial expressions denoting emotions, some experimental studies have focused on the inference of emotions using speech voices for patients with Parkinson’s disease (PD) and with eating disorders (ED) (Gray and Tickle-Degnen, 2010; Caglar-Nazali et al., 2014). Since these studies also involved discriminating emotions based on speech voices, it is clearly possible to examine the brain regions of patients using our experimental tasks (especially the voice-based Emotion N-back task). Our findings provide essential information contributing to the identification of biomarkers associated with difficulties inferring emotions based on vocal cues. In other words, there is a possibility that the emotion-specific brain activation observed in healthy participants may not be the same as that observed in the clinical group. In the future, it will be necessary to clarify the brain activation associated with the inference of emotions based on vocal cues in clinical groups.

For future clinical application, fNIRS offers unique merits in assessing auditory Emotion and Identification tasks. By using fNIRS, we were able to detect regions related to the inference of emotions with speech voices, controlling the effects of sounds other than the stimuli. While fMRI has the great advantage of being able to reveal and measure relevant regions, including deep cortical regions, it is difficult to completely eliminate the effects of non-stimulus noise during measurement. On the other hand, fNIRS can be used in a relatively quiet environment to control the influence of ambient sound on brain activation. Therefore, we believe that fNIRS is the most reliable method of examining the neural basis for the inference of emotions based on vocal cues. In this sense, our present study has clarified the cognitive processing associated with the inference of emotions based on vocal cues using fNIRS, which is of great clinical and academic significance.

## 5 Conclusion

We investigated the neural basis of working memory processing during auditory emotional judgment in healthy participants and revealed that different brain activation patterns can be observed between the two tasks (Emotion, Identity). During the Emotion task, there was significant activation of brain regions known to be part of an attentional network in auditory top-down processing. In addition, there was significant positive correlations between the improvement of behavioral performance and activated cortical regions including the right frontal region and the supramarginal gyrus. Therefore, the current study suggests that the cognitive demands of analyzing and processing the features of auditory information with auditory attention in order to make emotional judgments based solely on auditory information differ from those based on visual information such as facial expressions. Thus, we have provided an inclusive view of the cognitive mechanisms behind inferring the emotions of others based on analyses of fNIRS data and behavior data. In the future, it will be possible to apply our findings to the clinical diagnoses of some diseases associated with deficits of emotional judgment to contribute to early intervention and to providing criteria for selecting appropriate treatments.

## Data availability statement

The raw data supporting the conclusions of this article will be made available by the authors, without undue reservation.

## Ethics statement

The studies involving humans were approved by the Institutional Ethics Committee of Chuo University. The studies were conducted in accordance with the local legislation and institutional requirements. The participants provided their written informed consent to participate in this study.

## Author contributions

MK, HS, KO, and TH contributed to the conception and design of the study. HS, KO, and KN performed the experiments. SO, WK, KN, KO, and SN analyzed the data and performed the statistical analyses. YK supervised the statistical analysis plan. SO and KN wrote the manuscript. WK, MK, YK, and ID reviewed and edited the manuscript. WK and ID prepared the Supplementary material. ID supervised the study. All authors have reviewed the manuscript and approved the final version for publication.
