# Supplementary material for: Cerebral response to emotional working memory based on vocal cues: an fNIRS study
Source: Front Hum Neurosci. 2023 Dec 29;17:1160392. doi: 10.3389/fnhum.2023.1160392 (PMC10785654; doi:10.3389/fnhum.2023.1160392)
Supplement: Supplementary file 1 [file Data_Sheet_1.docx]

Supplementary Materials

Cerebral response to emotional working memory based on vocal cues: An fNIRS study

Saori Ohshima, Michihiko Koeda, Wakana Kawai, Hikaru Saito, Kiyomitsu Niioka, Koki Okuno, Sho Naganawa, Tomoko Hama, Yasushi Kyutoku, and Ippeita Dan*

*** Correspondence:** Corresponding Author: dan@brain-lab.jp

# Supplementary Tables

**Supplementary Table 1.**  Estimated most likely locations of all channels

| CH | MNI coordinates | | | | LPBA40 | Prob.  (%) | Brodmann area | Prob.  (%) |
| --- | --- | --- | --- | --- | --- | --- | --- | --- |
|  | x | y | z | *SD* (mm) |  |  |  |  |
| 1 | 63.0 | -37.3 | 51.3 | 9.0 | R SMG | 88 | SMG part of Wernicke's area (BA40) | 93 |
| 2 | 59.3 | -10.7 | 50.7 | 9.1 | R PoCG | 58 | M1 (BA4) | 49 |
| 3 | 49.0 | 15.3 | 52.3 | 9.1 | R MFG | 92 | DLPFC (BA9) | 81 |
| 4 | 32.7 | 33.7 | 52.0 | 9.4 | R MFG | 95 | DLPFC (BA9) | 66 |
| 5 | 13.3 | 44.3 | 54.0 | 9.3 | R SFG | 98 | DLPFC (BA9) | 65 |
| 6 | -10.7 | 44.3 | 53.7 | 9.5 | L SFG | 100 | DLPFC (BA9) | 64 |
| 7 | -31.7 | 31.7 | 52.0 | 9.7 | L MFG | 82 | DLPFC (BA9) | 64 |
| 8 | -47.7 | 14.3 | 50.7 | 9.2 | L MFG | 93 | DLPFC (BA9) | 69 |
| 9 | -58.3 | -11.3 | 48.7 | 9.2 | L PoCG | 75 | M1 (BA4) | 42 |
| 10 | -62.3 | -36.3 | 48.7 | 9.4 | L SMG | 100 | SMG part of Wernicke's area (BA40) | 89 |
| 11 | 64.7 | -48.3 | 39.7 | 10.0 | R AG | 78 | SMG part of Wernicke's area (BA40) | 90 |
| 12 | 68.0 | -20.3 | 38.7 | 8.6 | R SMG | 100 | S1 (BA1) | 54 |
| 13 | 61.0 | 7.3 | 38.3 | 8.5 | R PRG | 93 | Pre-SMA (BA6) | 81 |
| 14 | 47.7 | 33.3 | 39.7 | 8.5 | R MFG | 100 | pars triangularis Broca's area (BA45) | 39 |
| 15 | 26.3 | 51.3 | 41.3 | 9.0 | R MFG | 88 | DLPFC (BA9) | 92 |
| 16 | 1.0 | 55.0 | 41.0 | 9.3 | R SFG | 72 | DLPFC (BA9) | 96 |
| 17 | -23.3 | 50.7 | 41.3 | 9.7 | L MFG | 80 | DLPFC (BA9) | 90 |
| 18 | -45.3 | 33.3 | 38.3 | 9.0 | L MFG | 100 | pars triangularis Broca's area (BA45) | 48 |
| 19 | -59.0 | 7.7 | 36.7 | 9.0 | L PRG | 92 | Pre-SMA (BA6) | 77 |
| 20 | -66.0 | -19.3 | 36.7 | 8.9 | L SMG | 72 | S1 (BA2) | 49 |
| 21 | -65.0 | -46.3 | 37.3 | 9.5 | L SMG | 67 | SMG part of Wernicke's area (BA40) | 87 |
| 22 | 71.0 | -32.7 | 26.3 | 9.5 | R SMG | 64 | S1 (BA2) | 31 |
| 23 | 68.0 | -1.7 | 25.7 | 7.6 | R PoCG | 84 | Subcentral area (BA43) | 89 |
| 24 | 57.3 | 27.7 | 26.3 | 7.8 | R IFG | 87 | pars triangularis Broca's area (BA45) | 85 |
| 25 | 41.3 | 51.3 | 28.3 | 8.2 | R MFG | 94 | DLPFC (BA46) | 82 |
| 26 | 16.3 | 65.0 | 30.3 | 8.5 | R MFG | 88 | FPA (BA10) | 81 |
| 27 | -13.3 | 64.3 | 30.3 | 8.7 | L SFG | 61 | FPA (BA10) | 76 |
| 28 | -38.7 | 51.7 | 27.7 | 8.7 | L MFG | 100 | DLPFC (BA46) | 87 |
| 29 | -55.0 | 28.3 | 24.7 | 8.4 | L IFG | 66 | pars triangularis Broca's area (BA45) | 91 |
| 30 | -65.7 | -1.3 | 24.3 | 8.1 | L PoCG | 57 | Subcentral area (BA43) | 87 |
| 31 | -69.0 | -30.7 | 24.3 | 9.1 | L SMG | 61 | S1 (BA2) | 42 |
| 32 | 71.0 | -44.3 | 9.3 | 9.3 | R MTG | 76 | STG (BA22) | 77 |
| 33 | 71.0 | -13.7 | 9.3 | 8.2 | R STG | 83 | STG (BA22) | 99 |
| 34 | 63.0 | 18.3 | 12.7 | 7.7 | R PRG | 82 | pars opercularis part of Broca's area (BA44) | 54 |
| 35 | 52.0 | 45.7 | 12.7 | 7.4 | R IFG | 86 | pars triangularis Broca's area (BA45) | 56 |
| 36 | 30.7 | 66.7 | 14.7 | 7.9 | R MFG | 100 | FPA (BA10) | 99 |
| 37 | 2.7 | 68.7 | 15.3 | 9.0 | R SFG | 62 | FPA (BA10) | 100 |
| 38 | -27.3 | 66.7 | 14.3 | 8.6 | L MFG | 100 | FPA (BA10) | 96 |
| 39 | -49.0 | 46.7 | 11.7 | 8.3 | L IFG | 71 | pars triangularis Broca's area (BA45) | 52 |
| 40 | -59.7 | 19.7 | 11.3 | 8.0 | L IFG | 96 | pars opercularis part of Broca's area (BA44) | 43 |
| 41 | -67.7 | -12.7 | 7.3 | 8.7 | L STG | 66 | STG (BA22) | 94 |
| 42 | -70.0 | -41.7 | 7.3 | 8.8 | L STG | 51 | STG (BA22) | 79 |
| 43 | 73.0 | -25.7 | -6.7 | 7.8 | R MTG | 100 | MTG (BA21) | 100 |
| 44 | 66.0 | 2.3 | -5.7 | 7.6 | R STG | 83 | MTG (BA21) | 70 |
| 45 | 57.0 | 37.7 | -2.3 | 6.0 | R IFG | 99 | pars triangularis Broca's area (BA45) | 94 |
| 46 | 42.7 | 61.3 | -1.3 | 6.8 | R IFG | 89 | FPA (BA10) | 74 |
| 47 | 17.3 | 73.0 | 0.3 | 7.5 | R MFG | 100 | FPA (BA10) | 69 |
| 48 | -13.7 | 73.7 | -0.3 | 7.8 | L SFG | 61 | FPA (BA10) | 66 |
| 49 | -39.3 | 62.0 | -1.7 | 7.7 | L IFG | 56 | FPA (BA10) | 80 |
| 50 | -54.0 | 39.3 | -3.3 | 6.6 | L IFG | 89 | pars triangularis Broca's area (BA45) | 73 |
| 51 | -62.7 | 3.7 | -8.3 | 9.0 | L STG | 84 | MTG (BA21) | 62 |
| 52 | -71.0 | -23.3 | -9.3 | 7.8 | L MTG | 100 | MTG (BA21) | 100 |
| *Note.* AG: angular gyrus; DLPFC: dorsolateral prefrontal cortex; FPA: frontopolar cortex; IFG: inferior frontal gyrus; M1: primary motor cortex; MFG: middle frontal gyrus; MTG: middle temporal gyrus; PoCG: post central gyrus; Pre-SMA: pre-motor and supplementary motor cortices; PRG: precentral gyrus; S1: primary somatosensory cortex; SFG: superior frontal gyrus; SMG: supramarginal gyrus; and STG: superior temporal gyrus. | | | | | | | | |

**Supplementary Table 2.** One-sample *t*-tests assessing the size of inter-trial means of the oxy-Hb for the Emotion 2-back task

| CH | *M* | *SD* | *t* | *p* | *d* |
| --- | --- | --- | --- | --- | --- |
| 1 | 0.007 | 0.045 | 0.85 | .401 | 0.16 |
| 2 | 0.015 | 0.077 | 1.03 | .311 | 0.19 |
| 3 | 0.005 | 0.039 | 0.74 | .467 | 0.14 |
| 4 | 0.008 | 0.042 | 1.06 | .299 | 0.20 |
| 5 | 0.002 | 0.045 | 0.25 | .801 | 0.05 |
| 6 | 0.015 | 0.041 | 2.01 | .055 | 0.38 |
| 7 | 0.011 | 0.043 | 1.28 | .210 | 0.24 |
| 8 | -0.003 | 0.042 | -0.42 | .681 | -0.08 |
| 9 | 0.017 | 0.061 | 1.47 | .153 | 0.28 |
| 10 | 0.008 | 0.060 | 0.73 | .470 | 0.14 |
| 11 | 0.037 | 0.047 | 4.19 | <.001 | **0.79** |
| 12 | 0.029 | 0.108 | 1.44 | .163 | 0.27 |
| 13 | 0.054 | 0.090 | 3.20 | .004 | **0.60** |
| 14 | 0.015 | 0.050 | 1.59 | .122 | 0.30 |
| 15 | 0.008 | 0.052 | 0.77 | .449 | 0.15 |
| 16 | 0.009 | 0.063 | 0.79 | .439 | 0.15 |
| 17 | 0.010 | 0.051 | 1.05 | .304 | 0.20 |
| 18 | 0.019 | 0.047 | 2.21 | .035 | 0.42 |
| 19 | 0.010 | 0.048 | 1.13 | .267 | 0.21 |
| 20 | 0.017 | 0.075 | 1.22 | .234 | 0.23 |
| 21 | 0.035 | 0.050 | 3.65 | .001 | **0.69** |
| 22 | 0.052 | 0.123 | 2.22 | .035 | 0.42 |
| 23 | 0.067 | 0.196 | 1.80 | .083 | 0.34 |
| 24 | 0.044 | 0.071 | 3.26 | .003 | **0.62** |
| 25 | 0.026 | 0.061 | 2.25 | .033 | 0.43 |
| 26 | 0.003 | 0.062 | 0.23 | .820 | 0.04 |
| 27 | 0.006 | 0.061 | 0.53 | .597 | 0.10 |
| 28 | 0.026 | 0.058 | 2.34 | .027 | 0.44 |
| 29 | 0.024 | 0.050 | 2.53 | .018 | 0.48 |
| 30 | 0.042 | 0.088 | 2.53 | .018 | 0.48 |
| 31 | 0.039 | 0.082 | 2.54 | .017 | 0.48 |
| 32 | 0.076 | 0.147 | 2.72 | .011 | 0.51 |
| 33 | 0.049 | 0.190 | 1.37 | .183 | 0.26 |
| 34 | 0.057 | 0.152 | 1.99 | .057 | 0.38 |
| 35 | 0.028 | 0.075 | 2.00 | .055 | 0.38 |
| 36 | 0.024 | 0.067 | 1.89 | .069 | 0.36 |
| 37 | 0.013 | 0.088 | 0.77 | .446 | 0.15 |
| 38 | 0.014 | 0.073 | 0.98 | .337 | 0.18 |
| 39 | 0.035 | 0.047 | 3.99 | <.001 | **0.75** |
| 40 | 0.019 | 0.099 | 1.01 | .323 | 0.19 |
| 41 | 0.031 | 0.102 | 1.61 | .119 | 0.30 |
| 42 | 0.050 | 0.080 | 3.33 | .003 | **0.63** |
| 43 | 0.047 | 0.179 | 1.38 | .178 | 0.26 |
| 44 | 0.068 | 0.218 | 1.65 | .110 | 0.31 |
| 45 | 0.042 | 0.127 | 1.77 | .088 | 0.33 |
| 46 | 0.039 | 0.060 | 3.46 | .002 | **0.65** |
| 47 | 0.027 | 0.074 | 1.92 | .065 | 0.36 |
| 48 | 0.018 | 0.084 | 1.11 | .275 | 0.21 |
| 49 | 0.025 | 0.065 | 2.00 | .056 | 0.38 |
| 50 | 0.021 | 0.104 | 1.05 | .302 | 0.20 |
| 51 | 0.037 | 0.136 | 1.45 | .157 | 0.27 |
| 52 | 0.030 | 0.110 | 1.43 | .164 | 0.27 |
| *Note.* Channels with effect size, assessed using Cohen’s *d*, of 0.57 or more were defined as significantly activated channels. Cohen’s *d* of significantly activated channels are in boldface. | | | | | |

**Supplementary Table 3.** One-sample *t*-tests assessing the size of inter-trial means of the deoxy-Hb for the Emotion 2-back task

| CH | *M* | *SD* | *t* | *p* | *D* |
| --- | --- | --- | --- | --- | --- |
| 1 | 0.001 | 0.025 | 0.30 | .767 | 0.06 |
| 2 | -0.003 | 0.053 | -0.32 | .751 | -0.06 |
| 3 | -0.011 | 0.032 | -1.84 | .076 | -0.35 |
| 4 | 0.002 | 0.029 | 0.31 | .759 | 0.06 |
| 5 | 0.005 | 0.023 | 1.12 | .272 | 0.21 |
| 6 | 0.004 | 0.025 | 0.81 | .423 | 0.15 |
| 7 | 0.000 | 0.021 | 0.11 | .911 | 0.02 |
| 8 | -0.003 | 0.030 | -0.53 | .602 | -0.10 |
| 9 | 0.001 | 0.041 | 0.18 | .861 | 0.03 |
| 10 | 0.007 | 0.028 | 1.38 | .180 | 0.26 |
| 11 | -0.004 | 0.026 | -0.80 | .430 | -0.15 |
| 12 | 0.000 | 0.035 | 0.04 | .969 | 0.01 |
| 13 | 0.007 | 0.053 | 0.71 | .486 | 0.13 |
| 14 | -0.005 | 0.027 | -0.91 | .373 | -0.17 |
| 15 | -0.002 | 0.022 | -0.59 | .559 | -0.11 |
| 16 | 0.006 | 0.030 | 1.06 | .300 | 0.20 |
| 17 | 0.004 | 0.025 | 0.89 | .379 | 0.17 |
| 18 | -0.009 | 0.019 | -2.60 | .015 | -0.49 |
| 19 | 0.002 | 0.030 | 0.40 | .694 | 0.08 |
| 20 | 0.008 | 0.038 | 1.16 | .257 | 0.22 |
| 21 | 0.008 | 0.030 | 1.40 | .173 | 0.26 |
| 22 | -0.009 | 0.044 | -1.10 | .283 | -0.21 |
| 23 | -0.012 | 0.095 | -0.69 | .499 | -0.13 |
| 24 | -0.004 | 0.032 | -0.61 | .548 | -0.12 |
| 25 | -0.013 | 0.026 | -2.72 | .011 | -0.51 |
| 26 | 0.007 | 0.026 | 1.33 | .195 | 0.25 |
| 27 | 0.004 | 0.021 | 0.89 | .379 | 0.17 |
| 28 | -0.012 | 0.024 | -2.58 | .016 | -0.49 |
| 29 | -0.010 | 0.029 | -1.89 | .069 | -0.36 |
| 30 | -0.003 | 0.066 | -0.23 | .821 | -0.04 |
| 31 | 0.014 | 0.043 | 1.70 | .101 | 0.32 |
| 32 | -0.026 | 0.086 | -1.58 | .127 | -0.30 |
| 33 | -0.023 | 0.069 | -1.80 | .083 | -0.34 |
| 34 | -0.021 | 0.069 | -1.61 | .118 | -0.31 |
| 35 | -0.003 | 0.033 | -0.49 | .631 | -0.09 |
| 36 | -0.001 | 0.026 | -0.18 | .857 | -0.03 |
| 37 | 0.004 | 0.022 | 0.95 | .352 | 0.18 |
| 38 | 0.007 | 0.028 | 1.34 | .193 | 0.25 |
| 39 | -0.010 | 0.018 | -3.08 | .005 | **-0.58** |
| 40 | 0.002 | 0.051 | 0.17 | .870 | 0.03 |
| 41 | 0.001 | 0.058 | 0.10 | .923 | 0.02 |
| 42 | -0.001 | 0.035 | -0.08 | .936 | -0.02 |
| 43 | -0.030 | 0.079 | -2.02 | .053 | -0.38 |
| 44 | -0.020 | 0.108 | -1.00 | .327 | -0.19 |
| 45 | -0.004 | 0.038 | -0.60 | .557 | -0.11 |
| 46 | -0.007 | 0.024 | -1.43 | .165 | -0.27 |
| 47 | 0.000 | 0.020 | 0.09 | .925 | 0.02 |
| 48 | 0.001 | 0.016 | 0.47 | .641 | 0.09 |
| 49 | -0.004 | 0.021 | -1.05 | .302 | -0.20 |
| 50 | -0.008 | 0.041 | -1.01 | .321 | -0.19 |
| 51 | -0.006 | 0.065 | -0.46 | .651 | -0.09 |
| 52 | -0.004 | 0.047 | -0.40 | .689 | -0.08 |
| *Note.* Channels with effect size, assessed using Cohen’s *d*, of 0.57 or more were defined as significantly activated channels. Cohen’s *d* of significantly activated channels are in boldface. | | | | | |

**Supplementary Table 4.** One-sample *t*-tests assessing the size of inter-trial means of the oxy-Hb for the Identity 2-back task

| CH | *M* | *SD* | *t* | *p* | *d* |
| --- | --- | --- | --- | --- | --- |
| 1 | 0.008 | 0.054 | 0.83 | .414 | 0.16 |
| 2 | 0.022 | 0.098 | 1.21 | .237 | 0.23 |
| 3 | 0.022 | 0.054 | 2.16 | .040 | 0.41 |
| 4 | 0.007 | 0.054 | 0.71 | .484 | 0.13 |
| 5 | 0.008 | 0.043 | 1.00 | .326 | 0.19 |
| 6 | 0.011 | 0.038 | 1.51 | .143 | 0.29 |
| 7 | -0.003 | 0.056 | -0.29 | .770 | -0.06 |
| 8 | 0.000 | 0.057 | -0.02 | .985 | 0.00 |
| 9 | 0.022 | 0.073 | 1.61 | .118 | 0.31 |
| 10 | 0.026 | 0.067 | 2.08 | .048 | 0.39 |
| 11 | 0.013 | 0.042 | 1.61 | .119 | 0.30 |
| 12 | 0.022 | 0.077 | 1.49 | .147 | 0.28 |
| 13 | 0.028 | 0.084 | 1.79 | .084 | 0.34 |
| 14 | 0.017 | 0.050 | 1.77 | .088 | 0.33 |
| 15 | 0.008 | 0.048 | 0.89 | .382 | 0.17 |
| 16 | 0.004 | 0.054 | 0.42 | .675 | 0.08 |
| 17 | -0.003 | 0.064 | -0.22 | .826 | -0.04 |
| 18 | 0.014 | 0.049 | 1.49 | .149 | 0.28 |
| 19 | 0.020 | 0.050 | 2.15 | .040 | 0.41 |
| 20 | 0.048 | 0.080 | 3.16 | .004 | **0.60** |
| 21 | 0.035 | 0.056 | 3.34 | .002 | **0.63** |
| 22 | 0.035 | 0.087 | 2.13 | .042 | 0.40 |
| 23 | 0.043 | 0.128 | 1.78 | .087 | 0.34 |
| 24 | 0.016 | 0.086 | 0.96 | .345 | 0.18 |
| 25 | 0.022 | 0.052 | 2.23 | .035 | 0.42 |
| 26 | -0.006 | 0.051 | -0.63 | .533 | -0.12 |
| 27 | 0.004 | 0.062 | 0.35 | .728 | 0.07 |
| 28 | 0.013 | 0.066 | 1.06 | .298 | 0.20 |
| 29 | 0.024 | 0.062 | 2.06 | .050 | 0.39 |
| 30 | 0.074 | 0.104 | 3.77 | .001 | **0.71** |
| 31 | 0.070 | 0.110 | 3.36 | .002 | **0.63** |
| 32 | 0.054 | 0.076 | 3.81 | .001 | **0.72** |
| 33 | 0.021 | 0.107 | 1.04 | .309 | 0.20 |
| 34 | 0.032 | 0.119 | 1.44 | .162 | 0.27 |
| 35 | 0.020 | 0.051 | 2.11 | .044 | 0.40 |
| 36 | 0.026 | 0.057 | 2.47 | .020 | 0.47 |
| 37 | 0.021 | 0.076 | 1.43 | .164 | 0.27 |
| 38 | 0.018 | 0.078 | 1.25 | .223 | 0.24 |
| 39 | 0.019 | 0.064 | 1.55 | .132 | 0.29 |
| 40 | 0.048 | 0.095 | 2.66 | .013 | 0.50 |
| 41 | 0.070 | 0.140 | 2.64 | .014 | 0.50 |
| 42 | 0.075 | 0.114 | 3.48 | .002 | **0.66** |
| 43 | 0.007 | 0.096 | 0.41 | .683 | 0.08 |
| 44 | 0.014 | 0.138 | 0.54 | .594 | 0.10 |
| 45 | 0.022 | 0.092 | 1.26 | .219 | 0.24 |
| 46 | 0.043 | 0.059 | 3.92 | .001 | **0.74** |
| 47 | 0.030 | 0.064 | 2.52 | .018 | 0.48 |
| 48 | 0.031 | 0.076 | 2.14 | .042 | 0.40 |
| 49 | 0.022 | 0.067 | 1.76 | .090 | 0.33 |
| 50 | 0.019 | 0.063 | 1.62 | .118 | 0.31 |
| 51 | 0.048 | 0.149 | 1.71 | .098 | 0.32 |
| 52 | 0.048 | 0.145 | 1.75 | .092 | 0.33 |
| *Note.* Channels with effect size, assessed using Cohen’s *d*, of 0.57 or more were defined as significantly activated channels. Cohen’s *d* of significantly activated channels are in boldface. | | | | | |

**Supplementary Table 5.** One-sample *t*-tests assessing the size of inter-trial means of the deoxy-Hb for the Identity 2-back task

| CH | *M* | *SD* | *t* | *p* | *D* |
| --- | --- | --- | --- | --- | --- |
| 1 | 0.003 | 0.019 | 0.87 | .392 | 0.16 |
| 2 | -0.010 | 0.047 | -1.13 | .269 | -0.21 |
| 3 | -0.020 | 0.034 | -3.04 | .005 | **-0.57** |
| 4 | 0.001 | 0.026 | 0.25 | .802 | 0.05 |
| 5 | 0.003 | 0.020 | 0.79 | .438 | 0.15 |
| 6 | 0.006 | 0.024 | 1.29 | .209 | 0.24 |
| 7 | 0.007 | 0.023 | 1.68 | .105 | 0.32 |
| 8 | -0.002 | 0.038 | -0.32 | .748 | -0.06 |
| 9 | 0.005 | 0.049 | 0.49 | .630 | 0.09 |
| 10 | -0.004 | 0.026 | -0.89 | .383 | -0.17 |
| 11 | 0.005 | 0.022 | 1.23 | .229 | 0.23 |
| 12 | 0.005 | 0.032 | 0.83 | .416 | 0.16 |
| 13 | -0.005 | 0.039 | -0.72 | .480 | -0.14 |
| 14 | -0.008 | 0.019 | -2.14 | .042 | -0.40 |
| 15 | 0.000 | 0.027 | 0.10 | .921 | 0.02 |
| 16 | 0.008 | 0.040 | 1.05 | .302 | 0.20 |
| 17 | 0.014 | 0.031 | 2.37 | .025 | 0.45 |
| 18 | -0.008 | 0.023 | -1.96 | .061 | -0.37 |
| 19 | -0.007 | 0.029 | -1.19 | .245 | -0.22 |
| 20 | 0.002 | 0.044 | 0.30 | .767 | 0.06 |
| 21 | 0.010 | 0.026 | 2.12 | .043 | 0.40 |
| 22 | 0.001 | 0.038 | 0.16 | .876 | 0.03 |
| 23 | 0.002 | 0.080 | 0.14 | .887 | 0.03 |
| 24 | -0.004 | 0.035 | -0.59 | .561 | -0.11 |
| 25 | -0.013 | 0.028 | -2.33 | .027 | -0.44 |
| 26 | 0.009 | 0.034 | 1.38 | .179 | 0.26 |
| 27 | 0.011 | 0.035 | 1.70 | .101 | 0.32 |
| 28 | -0.005 | 0.033 | -0.85 | .400 | -0.16 |
| 29 | -0.016 | 0.023 | -3.70 | .001 | **-0.70** |
| 30 | -0.007 | 0.079 | -0.44 | .665 | -0.08 |
| 31 | 0.016 | 0.069 | 1.25 | .223 | 0.24 |
| 32 | 0.000 | 0.039 | 0.05 | .958 | 0.01 |
| 33 | -0.013 | 0.049 | -1.37 | .181 | -0.26 |
| 34 | -0.006 | 0.061 | -0.50 | .621 | -0.09 |
| 35 | -0.002 | 0.032 | -0.31 | .758 | -0.06 |
| 36 | 0.001 | 0.031 | 0.13 | .901 | 0.02 |
| 37 | 0.010 | 0.032 | 1.64 | .112 | 0.31 |
| 38 | 0.016 | 0.033 | 2.50 | .019 | 0.47 |
| 39 | -0.011 | 0.026 | -2.30 | .029 | -0.43 |
| 40 | -0.009 | 0.059 | -0.83 | .412 | -0.16 |
| 41 | -0.004 | 0.079 | -0.26 | .801 | -0.05 |
| 42 | 0.000 | 0.052 | 0.04 | .971 | 0.01 |
| 43 | -0.009 | 0.045 | -1.05 | .302 | -0.20 |
| 44 | -0.014 | 0.057 | -1.33 | .195 | -0.25 |
| 45 | -0.004 | 0.053 | -0.44 | .667 | -0.08 |
| 46 | -0.010 | 0.038 | -1.44 | .161 | -0.27 |
| 47 | -0.001 | 0.022 | -0.35 | .733 | -0.07 |
| 48 | 0.005 | 0.022 | 1.07 | .293 | 0.20 |
| 49 | 0.002 | 0.032 | 0.27 | .792 | 0.05 |
| 50 | -0.010 | 0.044 | -1.26 | .218 | -0.24 |
| 51 | -0.012 | 0.077 | -0.84 | .410 | -0.16 |
| 52 | -0.010 | 0.072 | -0.76 | .452 | -0.14 |
| *Note.* Channels with effect size, assessed using Cohen’s *d*, of 0.57 or more were defined as significantly activated channels. Cohen’s *d* of significantly activated channels are in boldface. | | | | | |

# Supplementary Figures


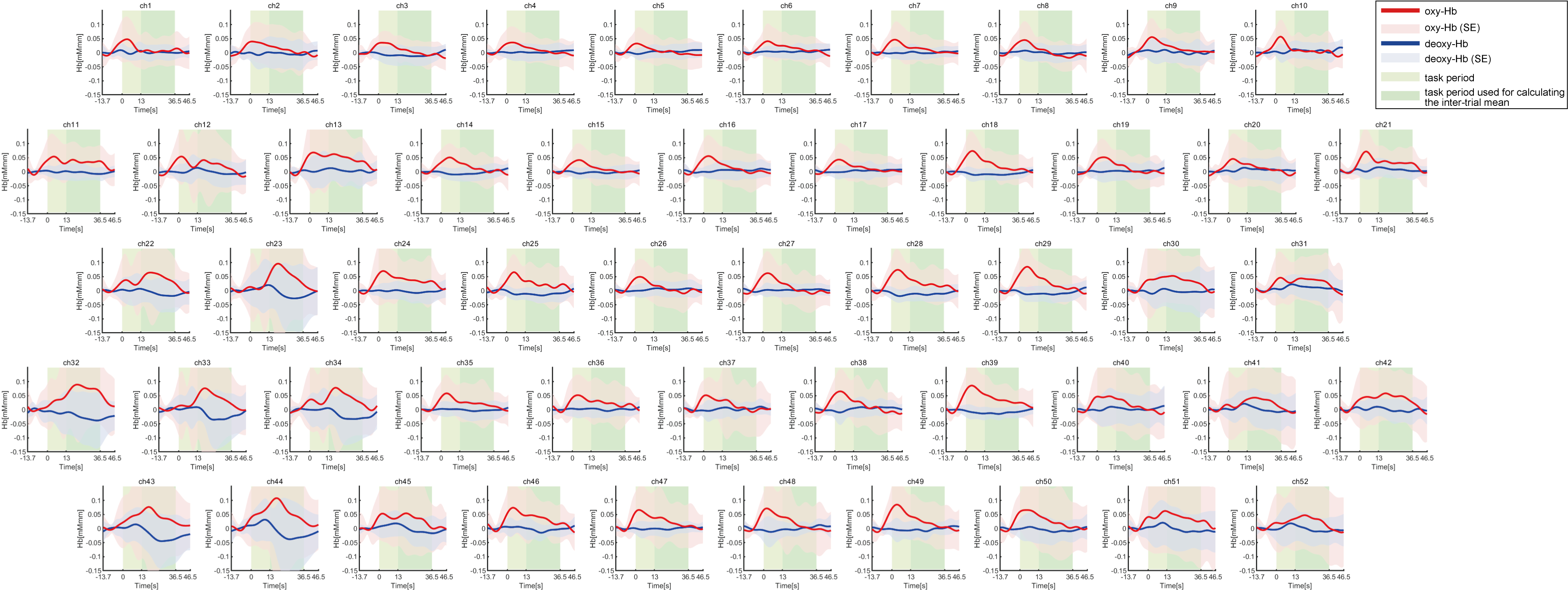


**Supplementary Figure 1.** Averaged oxy-Hb and deoxy-Hb time-series data for the Emotion 2-back task


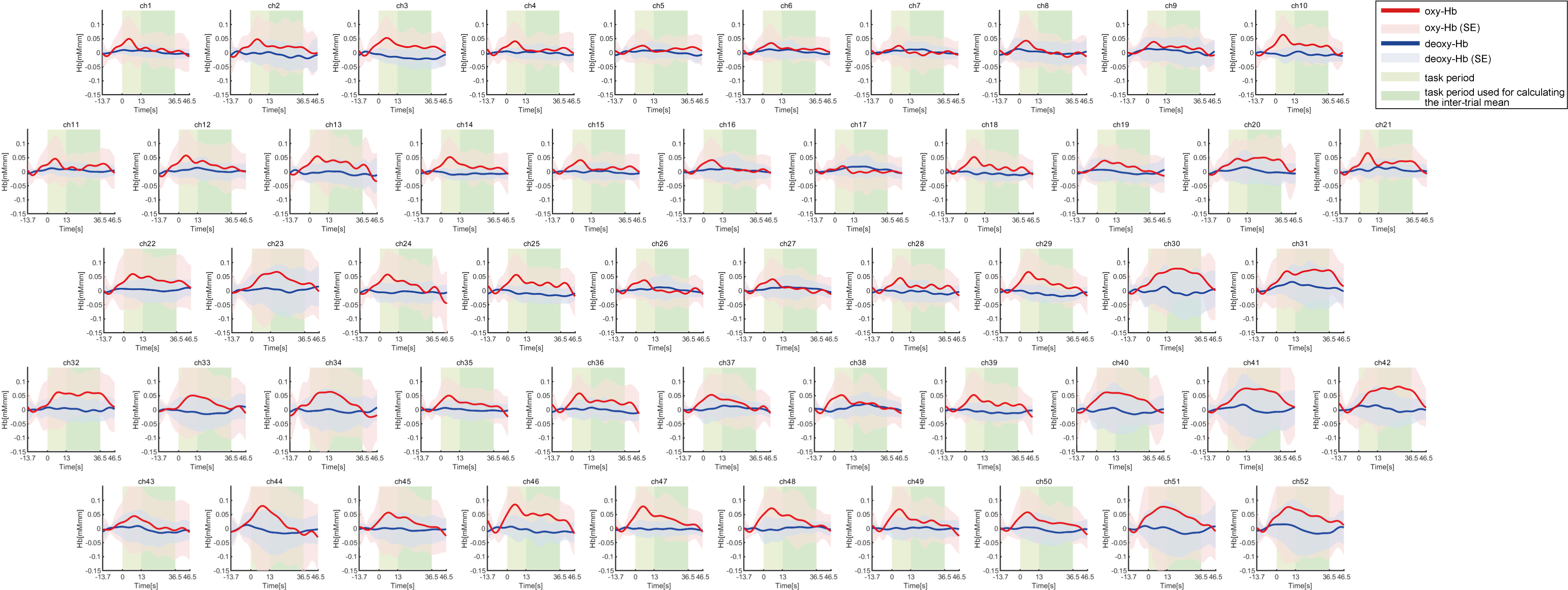


**Supplementary Figure 2.** Averaged oxy-Hb and deoxy-Hb time-series data for the Identity 2-back task
